# Comparative analysis of CNN–LSTM and CNN–BiLSTM hybrid deep learning models for solar radiation prediction

**DOI:** 10.1038/s41598-026-59621-5

**Published:** 2026-06-28

**Authors:** Yogita Yashveer Raghav, Parveen Kumari, Prashanth kumar katta, Sakshi Kathuria, Manisha Mudgal, M. Prabhakar, Mohamed Yusuf

**Affiliations:** 1 Centre of Excellence-Cloud Computing, School of Engineering & Technology, K.R. Mangalam University, Gurugram, India; 2 Department of Artificial Intelligence and Machine Learning, Dronacharya College of Engineering, Gurugram, India; 3 Department of Restorative Dental Sciences, College of Dentistry, King Faisal University, Al-Ahsa, Kingdom of Saudi Arabia; 4 Amity School of Engineering and Technology, Amity University, Gurugram, Haryana India; 5 Computer Science Engineering, Shri Vishwakarma Skill University, Haryana, India; 6 Department of Computer Science and Engineering, Dayananda Sagar University, Bangalore, India; 7 Department of Peace and Development Studies, Njala University, Bo Campus –18, Bo, Sierra Leone

**Keywords:** Solar radiation forecasting, Hybrid deep learning, CNN–LSTM, CNN–BiLSTM, Multivariate climate variables, Energy science and technology, Engineering, Mathematics and computing

## Abstract

Solar radiation forecasting is a complex task since the radiation signal is nonlinear, intermittent and is significantly influenced by meteorological variability, which makes it vital for PV planning, renewable energy planning and stability of the smart grid. In this work, a replicable comparison between CNN–LSTM and CNN–BiLSTM models for one-step ahead solar clearness-index forecasting based on multivariate climate variables from NASA POWER dataset for Delhi, India, is presented. Under identical preprocessing, windowing, chronological splitting, and training conditions, CNN–LSTM achieved MAE = 0.0880, RMSE = 0.1100, R2 = 0.3100, EVS = 0.3154, WI = 0.6317, and APB = 1.89%, whereas CNN–BiLSTM obtained MAE = 0.1015, RMSE = 0.1224, R2 = 0.1456, EVS = 0.1998, WI = 0.5261, and APB = 5.98%. The Skill Scores shown and the negative values for direct clearness-index prediction do not imply that the persistence reference was unattainable, but rather reveal that the results are a controlled model-to-model comparison and not evidence of state-of-the-art superiority. Reconstructed all-sky irradiance produced stronger agreement with observations (MAE = 0.4353, RMSE = 0.5417, R2 = 0.7884, EVS = 0.7965, WI = 0.9299, and APB = 3.90%). The main task of CNN–LSTM is to provide a practical balance between accuracy and efficiency in this experimental context, and further testing with other locations, more powerful baselines and probabilistic forecasting techniques is needed.

## Introduction

 Accurate forecasting of solar radiation (solar irradiance) is critical for reliable photovoltaic (PV) integration, unit commitment and dispatch planning, reserve allocation, energy trading, and overall smart grid stability. However, irradiance forecasting remains challenging because solar signals ex- hibit strong nonlinearity, intermittency, and nonstationary behavior caused by cloud dynamics and rapidly changing meteorological conditions. Recent reviews emphasize that deep learning (DL) models consistently improve forecast accuracy compared with classical statistical and machine- learning baselines when (i) multivariate meteorological predictors are used and (ii) temporal de- pendencies are learned directly from sequences^1–3^.

Among DL approaches, recurrent neural networks (RNNs) and LSTM variants are widely adopted for solar and PV forecasting because they capture temporal dependencies from historical time windows and can generalize across different horizons^4–6^. Nevertheless, purely recurrent architectures may under-represent localized fluctuations and cross-variable interactions that occur at short time scales. To address this limitation, hybrid CNN–RNN models have become popular: CNN blocks act as feature extractors over multivariate sequences, while LSTM/GRU modules model longer-term temporal structure^5,7–9^. This hybrid design is attractive for operational forecasting because it can improve robustness under variable weather regimes without relying on highly engineered handcrafted features.

Bidirectional recurrent units (e.g., BiLSTM) have also been investigated to enrich contextual learning by leveraging sequence information from both directions within the input window, which may help in cases where the input contains complex patterns driven by multi-day transitions^10^. In parallel, research increasingly reports that performance depends strongly on site-specific climate, horizon (intra-hour, hourly, daily), and the chosen feature set and preprocessing protocol; consequently, fair benchmarking under a consistent evaluation pipeline is necessary to draw reliable conclusions^1,2,6^.

More recently, attention-based mechanisms and Transformer-style models have been introduced to improve long-range dependency learning and to enhance feature selection across time^11–13^. For very short-term horizons, multimodal pipelines that combine irradiance history with visual cues (e.g., ground-based sky images) have reported further gains, suggesting that cloud motion information can complement meteorological predictors^14,15^. Despite these advances, many studies still report results under heterogeneous settings (e.g., different splits, unequal feature availability, and inconsistent hyperparameter tuning), making it difficult to isolate the benefit of a specific architectural choice.

Considering these challenges, this study implements a controlled comparison between CNN–LSTM and CNN–BiLSTM network models to forecast solar radiation in multivariate and solar applications. The two architectures of CNN layers and LSTM-based layers are appropriate for the current task, as CNN layers can capture local temporal patterns and interactions among the variables in climate sequences, while LSTM-based layers would capture the temporal dependencies that are needed for one-step-ahead forecasting^7,8,10^. The unidirectional and bidirectional recurrent variants are compared with the same CNN front-end to eliminate the effect of sequence encoding. This study deals with the main challenges such as nonlinear irradiance behaviour, weather-driven sudden changes of state, comparability of previous studies and reproducible evaluation of the models. This is not a novel neural network design, but a clear and consistent benchmarking process, as it is based on the same network preprocessing, the same temporal variables, the same time-splitting and the same relative improvement discussion and uncertainty discussion, reporting the results in a way that is easy to compute. In the scenario reported, the adopted method, CNN–LSTM, achieves marginally higher predictive accuracy at the same inference cost as CNN–BiLSTM; consequently, the former is more suitable for deployment and reproducible solar-radiation forecasting applications where efficiency and reproducibility are of critical importance.

## Literature review

Solar irradiance (solar radiation) forecasting is a foundational requirement for PV generation scheduling, grid balancing, reserve planning, and electricity-market participation. The task is inherently difficult because surface irradiance is driven by fast cloud evolution and complex atmo- spheric processes (e.g., aerosols, humidity, and wind-driven transitions), producing intermittent, nonlinear, and nonstationary time-series. Across the literature, forecasting approaches are com- monly grouped into (i) physical/NWP-based methods, (ii) statistical models (e.g., ARIMA-family), and (iii) data-driven machine-learning (ML) and deep learning (DL) models. Recent reviews consistently report that DL methods are competitive—often superior— when sufficient historical coverage and multivariate meteorological predictors are available, and when evaluation is per- formed chronologically without leakage^1,3,16^. However, reported performance gains can vary substantially across regions, forecast horizons, data quality, and preprocessing choices, which motivates controlled experimentation and transparent reporting^1,16,17^.

### Sequence learning With RNN/LSTM models

Early and continuing DL work shows that recurrent architectures are well-suited for irradiance/PV forecasting because they learn temporal dependencies and persistence patterns directly from his- torical sequences. For example, LSTM models have been shown to outperform several classical baselines for day-ahead GHI forecasting, including when satellite-derived inputs are used^18^. Beyond standard univariate setups, multivariate LSTM pipelines that incorporate weather variables are commonly reported to improve stability under changing regimes (clear, cloudy, and transitional conditions), although accuracy remains sensitive to window length and feature selection^17,19^.

### CNN–RNN hybrid forecasting

Hybrid CNN–RNN architectures are widely used because they combine complementary modeling strengths. Convolutional layers learn local temporal structures and cross-variable interactions from multivariate signals, while recurrent layers (e.g., LSTM/GRU) capture sequential dependencies and longer-term memory. In solar forecasting, hybrid CNN–LSTM-type pipelines have repeatedly demonstrated improved accuracy and robustness compared with standalone CNN or standalone RNN models, particularly when meteorological inputs are included and the evaluation spans mul- tiple seasons and sky conditions^8,20,21^. In practice, the effectiveness of such hybrids is highly influenced by design choices such as look-back window length, feature scaling strategy, and the balance between convolutional capacity and recurrent capacity; these factors can directly affect error stability and generalization^21,22^. Multi-step or multi-horizon forecasting studies further suggest that deeper temporal modeling can help, but the benefit depends on data resolution and the frequency of abrupt irradiance changes^4,5^.

### Bidirectional recurrent units and decomposition-based methods

Bidirectional recurrent networks (BiLSTM) have been explored to enrich sequence representation by incorporating contextual cues from both directions within the input window. Some works report improved learning under highly variable climates and longer horizons, while other studies observe only marginal gains relative to standard LSTM, depending on the forecasting protocol and feature quality^23,24^. Alongside architectural choices, decomposition and reconstruction strategies remain popular for addressing nonstationarity and noise. For instance, wavelet- or CEEMDAN- style decomposition combined with deep sequence learners has been used to separate frequency components or intrinsic modes before forecasting, which can reduce error by simplifying the learning task^25–27^. More recent hybrid designs explicitly combine decomposition, CNN feature extraction, and attention-enhanced recurrent modules to improve robustness under complex variability^28^.

### Attention mechanisms, transformers, and feature-enhanced models

Attention and self-attention mechanisms are increasingly adopted to emphasize the most informative time steps and improve robustness during rapid weather transitions^22,29^. Transformer-style architectures have also been introduced for irradiance forecasting to model long-range dependencies and to support multi-step prediction, especially when long sequences are used^11,30^. Recent reviews of PV/irradiance forecasting report a clear trend toward attention/Transformer families, while also noting that consistent benchmarks and fair protocols are still lacking across studies^31,32^. Additionally, Transformer-infused recurrent hybrids have been proposed to blend recurrent inductive bias with attention capacity^33^. In parallel, feature-enhanced CNN–LSTM variants that integrate attention and similar-day selection have demonstrated gains in next-day prediction by aligning meteorological regimes and selectively weighting temporal contexts^34^.

Recent forecasting studies outside solar irradiance also emphasize the value of rigorous benchmarking, hybrid architectures, prediction intervals, and multiple evaluation criteria. Examples include AI-based water-level prediction, exchange-rate forecasting, electric-load forecasting with hybrid SVR and metaheuristic methods, and transformer-based electricity-demand prediction with local climate variables^35–41^. These studies are relevant to the present work because they highlight the need to report complementary accuracy, robustness, and uncertainty measures rather than relying on a single metric.

### Very short-term forecasting and evaluation practices

For very short horizons, cloud dynamics and abrupt atmospheric changes dominate irradiance variability. Minute-level and intra-hour studies indicate that combining historical irradiance with meteorological cues and sequence models can improve ultra-short-term prediction accuracy, al- though sensitivity to sensor noise and rapid regime shifts remains challenging^42^. More broadly, comparative studies emphasize that model ranking is highly sensitive to geography, horizon, and feature availability, motivating rigorous evaluation under consistent preprocessing, chronological splits, and standardized metrics such as MAE, RMSE, and *R*^2^^16,43,44^. Recent guidance also stresses reproducible training protocols (fixed seeds, reported callbacks, and transparent hyper- parameter settings) and multiple error measures to capture different aspects of predictive quality^3,17^.

### Relevance to the proposed approach

Ganvir et al. (2024) explored global horizontal irradiance prediction using explainable artificial intelligence, emphasizing interpretability in solar forecasting^45^, while Gupta et al. (2024) improved irradiance estimation by coupling CNN with wavelet transform, demonstrating that hybrid deep learning and signal decomposition can reduce error under atmospheric variability^46^.The approach adopted in this study aligns with these directions by (i) constructing multivariate input sequences using a fixed look-back window, (ii) applying CNN modules to learn local tem- poral patterns and cross-variable interactions, (iii) using LSTM/BiLSTM layers to model temporal dependency, and (iv) evaluating performance with MAE, RMSE, and *R*2 for direct benchmarking against related work^1,8,20,23^. Additionally, reconstructing all-sky irradiance using a clearness-index-style relation is consistent with broader efforts that separate clear-sky effects from atmospheric variability to improve stability and interpretability^28,42,44^.

### Problem statement and research gap

Reliable solar radiation forecasting is essential for PV scheduling and smart-grid stability, yet high accuracy is difficult because irradiance is nonlinear, intermittent, and strongly weather-dependent. While many studies propose DL models, several gaps remain. First, some approaches use limited feature sets (e.g., only irradiance history or a small subset of meteorological variables), which may not fully represent the multivariate drivers of irradiance^3,16^. Second, comparisons across architectures are often not strictly controlled because preprocessing, window lengths, data splits, and hyperparameter tuning differ across studies, making it hard to attribute improvements to the model itself^1,43^. Third, key design decisions such as windowing strategy, normalization, and engineered temporal statistics are not always reported, reducing reproducibility and limiting fair benchmarking^8,17,21^. Finally, many experiments are performed on a single site or limited time span, which can restrict generalization and practical deployment^16,19^.

A controlled comparison between CNN–LSTM and CNN–BiLSTM is therefore needed. Both hybrids are popular because CNN layers learn informative local patterns from multivariate se- quences, while recurrent layers model temporal structure. However, BiLSTM does not consistently outperform standard LSTM in forward-looking forecasting, and its impact may depend on data characteristics, sequence length, and protocol choices^23,24^. Benchmarking both models under the same dataset, feature set, and evaluation pipeline helps identify whether bidirectionality yields measurable gains in this setting.

### Why CNN–LSTM and CNN–BiLSTM were chosen

The CNN–LSTM model and the CNN–BiLSTM model were the first two models selected due to two reasons. First, both the architectures are prevalent hybrid architectures for multivariate solar forecasting tasks as CNN could extract local temporal patterns while the LSTM-based units could learn sequential dependencies. Second, by training only one CNN per type and architecture (unidirectional vs. bidirectional), the study can control for the value added by bidirectional recurrence. In future work, a more comprehensive benchmarking scope can be expanded by implementing a CNN–GRU baseline and using an independent recurrent baseline model.

### Contributions

This study has contributed a deployable and reproducible comparative model for the prediction of solar radiation in the climatic context of Delhi. It’s not about introducing a completely new neural architecture, but rather about the novelty of controlled benchmarking, transparent preprocessing, practical efficiency analysis, feature-relevance interpretation, uncertainty discussion, and clear reporting of model trade-offs.

- The study reveals several critical forecasting issues, such as the nonlinearity, intermittency due to weather, risk of data leakage, high sensitivity to hyperparameters, and lack of comparability between different heterogenous protocols.
- A multivariate input representation is created by stacking meteorological variables along with calendar variables, lagged clearness-index values, and rolling statistics to capture the seasonality, recent and local variability patterns^8,27^.
- Using the same pre-processing, windowing, chronological split, optimizer, callbacks and evaluation settings, CNN–LSTM and CNN–BiLSTM models are trained to provide a fair comparison of the effect of recurrent design^20,21,23^.
- To present a comprehensive evaluation of the performance, standard regression metrics are used along with computational efficiency, relative improvement, approximate uncertainty assessment and discussion of feature-relevance^1,16,43^.
- A reproducible methodology is given from data acquisition to data cleaning, data scaling, windowing of sequences, reporting of hyperparameters, splitting of data chronologically into train, validation and test sets, to analysis of model comparisons^3,17^. It is a reproducible comparison between CNN–LSTM and CNN–BiLSTM where the same data, feature-engineering steps, sequence construction, training protocol and evaluation metrics are employed to draw a fair conclusion on the accuracy of prediction and the practical efficiency.

## Dataset and preprocessing

### Dataset source and study region

 Daily solar and meteorological time-series were collected from the NASA POWER (Prediction Of Worldwide Energy Resources) web API for a single reference point representing Delhi, India (latitude 28.6139◦, longitude 77.2090◦). The multi-year period from **01-Jan-2018** to **31-Dec- 2024** was selected to provide a sufficiently large sample base for training and evaluation, and to capture inter-annual seasonal variability that is important for generalization in solar forecasting^1,3,8,16,21^. The clearness index, ALLSKY KT, is used as the primary target because it reflects atmospheric attenuation effects and is frequently adopted in irradiance modeling. In addition, clear-sky surface shortwave downward irradiance (CLRSKY SFC SW DWN) is retrieved to support a clear-sky-guided reconstruction of all-sky irradiance, which has been reported to improve stability by separating deterministic clear-sky behavior from weather-driven variability^8,23,42^.

**Fig. 1:**
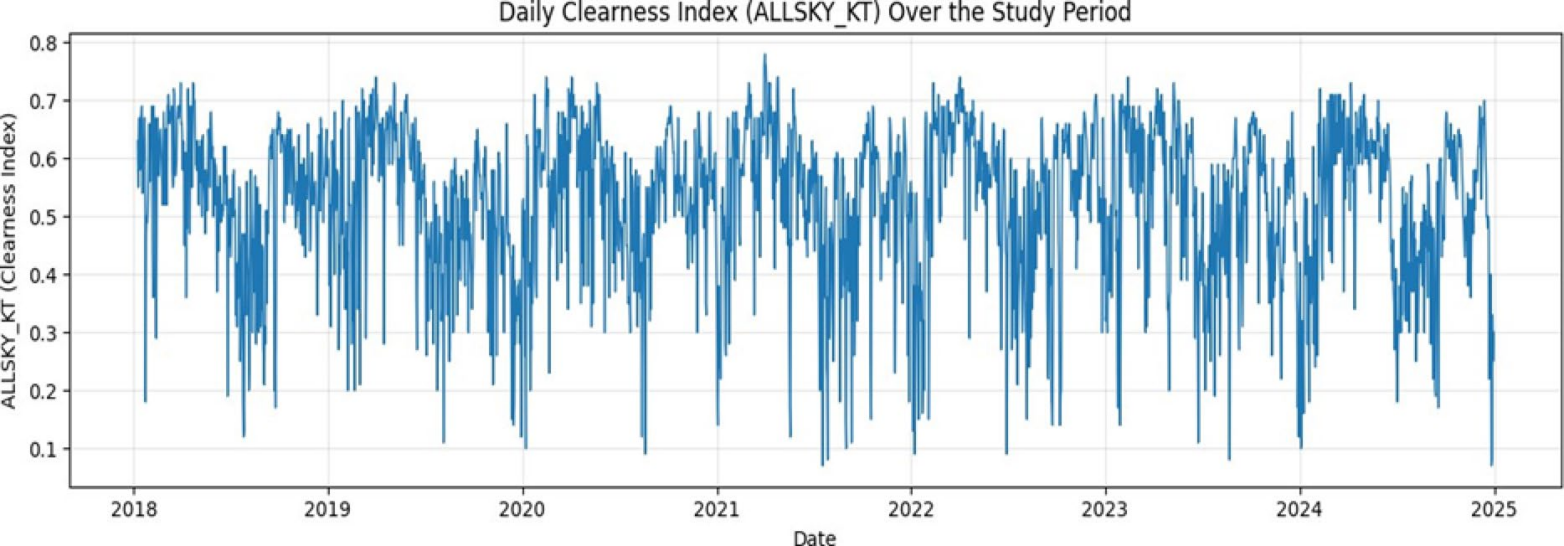
Daily clearness index (ALLSKY KT) over the study period (2018–2024).

Figure 1 illustrates the full-period daily evolution of the clearness index (ALLSKY KT) from 2018 to 2024. The plot shows clear seasonal oscillations with recurring peaks during high-transparency periods and pronounced dips associated with cloudy or monsoon-like conditions. Short-lived sharp drops and recoveries are also visible, highlighting the intermittency and fast atmospheric transitions that make irradiance-related forecasting difficult. This observed variability motivates the use of multivariate inputs and temporal modeling so that models can learn both long-term seasonal behavior and short-term fluctuations within a unified forecasting framework^1,16^.

### Variables used

The final dataset contains the target variable ALLSKY KT along with multiple meteorological pre- dictors: CLRSKY SFC SW DWN, T2M, T2M MAX, T2M MIN, RH2M, PS, PRECTOTCORR, and WS2M. These

variables are commonly reported as influential drivers of short-term solar variability because they represent temperature conditions, humidity effects, air pressure changes, precipitation-related dy- namics, and wind-linked transitions that correlate with cloud movement and atmospheric state^1,4,16,17^. After retrieval, the index was parsed into a date-time format and the table was ordered chronologically to preserve temporal consistency, which is essential for sequence modeling and avoids leakage in forecasting evaluation^3,4,27^.

### Data cleaning and consistency checks

NASA POWER encodes missing values using sentinel entries; therefore, all occurrences of -999 were replaced with NaN. Rows containing incomplete values were removed to prevent invalid inputs from propagating through the pipeline. Each column was then explicitly converted to numeric form using coercion to handle unexpected non-numeric entries, followed by a final pass to drop any remaining missing values. Such cleaning and type-consistency checks are recommended for reliable DL training, especially when building hybrid CNN–RNN models that can be sensitive to outliers and invalid values^3,16,21^.

### Feature engineering

 To enhance seasonal representation, two calendar-based predictors were added: day of year and month. Short-term memory was incorporated by constructing lagged versions of the target: KT lag 1, KT lag 3, and KT lag 7. Rolling statistics were also computed to summarize recent behavior and volatility: KT roll mean 7 and KT roll std 7. These temporal descriptors are commonly used to improve learning under nonstationary solar signals and to provide models with local trend/variance cues that pure sequence models may not capture efficiently^22,25–27^. Since lagging and rolling operations introduce undefined values at the beginning of the series, the corresponding rows were removed after feature generation to preserve alignment between inputs and outputs^5^.

### Normalization

All predictors were scaled to the range [0, 1] using Min–Max normalization. Independent scalers were fitted for the input feature matrix and the target variable to reduce leakage risk and enable correct inverse transformation during evaluation. Normalization is widely used in solar forecasting.

pipelines because it improves numerical stability and supports more consistent convergence of gradient-based training for deep hybrid architectures^8,17,47^.

### Sequence construction (Windowing)

The forecasting problem was formulated as a supervised sequence task using a fixed look-back window of **WINDOW = 90** days. For each time step *t*, the input sample is a multivariate sequence covering the previous 90 days of engineered predictors, while the output corresponds to the target clearness index at *t* + 1 (one-step-ahead prediction). This sliding-window construction produces tensors of the form (samples, window, features), which is the standard format for CNN–.

LSTM/CNN–BiLSTM training and is commonly adopted in recent hybrid forecasting studies^4,8,20,21^.

### Train–test split and validation strategy

 To avoid temporal leakage, sequences were split chronologically without shuffling. The earliest 80% of samples were used for training and the remaining 20% were reserved for testing. During training, 10% of the training subset served as a validation split (validation split = 0.1) to monitor generalization and support early stopping and learning-rate scheduling. Chronological evaluation and multi-metric reporting are widely recommended for realistic solar forecasting benchmarks, because random shuffling can inflate performance by leaking future structure into training^3,16,43,44^. For reproducibility, random seeds were fixed for both NumPy and TensorFlow, consistent with best practices in deep learning experimentation^17^.

### All-sky irradiance reconstruction for evaluation

Beyond clearness index prediction, all-sky irradiance was reconstructed using the clear-sky com- ponent:

$$AllSky(t) = ClearSky(t) \times \widehat{{KT}}(t),$$

where CLRSKY SFC SW DWN is the clear-sky irradiance and *KT* (*t*) is the predicted clearness index. Clear-sky values were aligned with test targets by accounting for the look-back window offset so that

reconstruction and error computation were performed on matched timestamps. Clear-sky-guided reconstruction is consistent with the general direction of improving irradiance predictability by separating clear-sky effects from atmospheric variability and has been reported to stabilize learning in practical forecasting settings^13,23,42,48,49^.

**Fig. 2:**
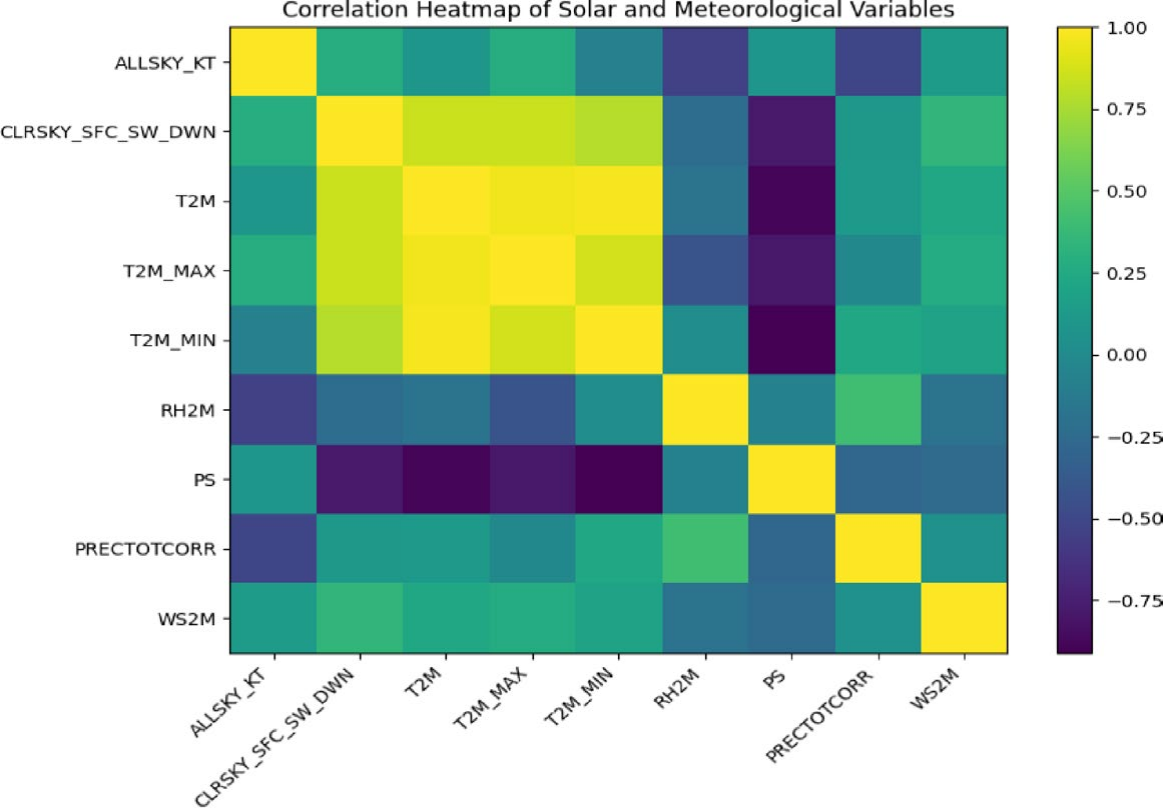
Correlation heatmap of ALLSKY KT and selected meteorological predictors.

Figure 2 provides a correlation heatmap for ALLSKY KT and the major meteorological predictors used in this work. The visualization highlights how solar clearness is related to radiation and weather variables, and it also reveals inter-dependencies among predictors (e.g., temperature and humidity- related variables), which is important when designing multivariate learning models. Stronger associations between ALLSKY KT and radiation-related variables (such as CLRSKY SFC SW DWN) are expected because clear-sky forcing provides a baseline envelope for available solar energy. In contrast, weaker or mixed correlations with humidity, precipitation, and wind variables indicate that atmospheric attenuation is influenced by complex nonlinear interactions rather than single- variable effects, motivating the use of hybrid deep models capable of learning multi-factor temporal relationships^1,8,16^. Additionally, visible correlations among meteorological inputs suggest potential redundancy, which supports the use of CNN layers to learn compact feature representations before temporal modeling^8,21^.

## Methodology and proposed framework

### Forecasting setup and input representation

Solar forecasting is formulated as a one-step-ahead regression task. At each time index *t*, a multivariate sequence $X_{t} \in \,R^{{w \times F}}$ is constructed, where *w* denotes the look-back window length and *F* represents the number of input variables. The objective is to learn a mapping *f* (·) that predicts the next-step clearness index:

$$\hat{y}_{{t + 1}} = f({\mathbf{X}}_{t} ),$$

where y_t+1_ is the ground-truth target and y’_t+1_ is the model prediction. This formulation is widely adopted in solar and PV forecasting because it supports chronological evaluation and direct inte-

gration into operational decision-making pipelines^3,50,51^. Following standard deep learning practice for time-series, the input is organized as a 3D tensor (samples, window, features)^19,20,50^. In this study, *w* = 90 days is used, producing tensors of shape (samples, 90, F). The selected window length is intended to capture short-term persistence as well as seasonal and meteorological transitions that may influence irradiance attenuation^4,17^.

**Fig. 3:**
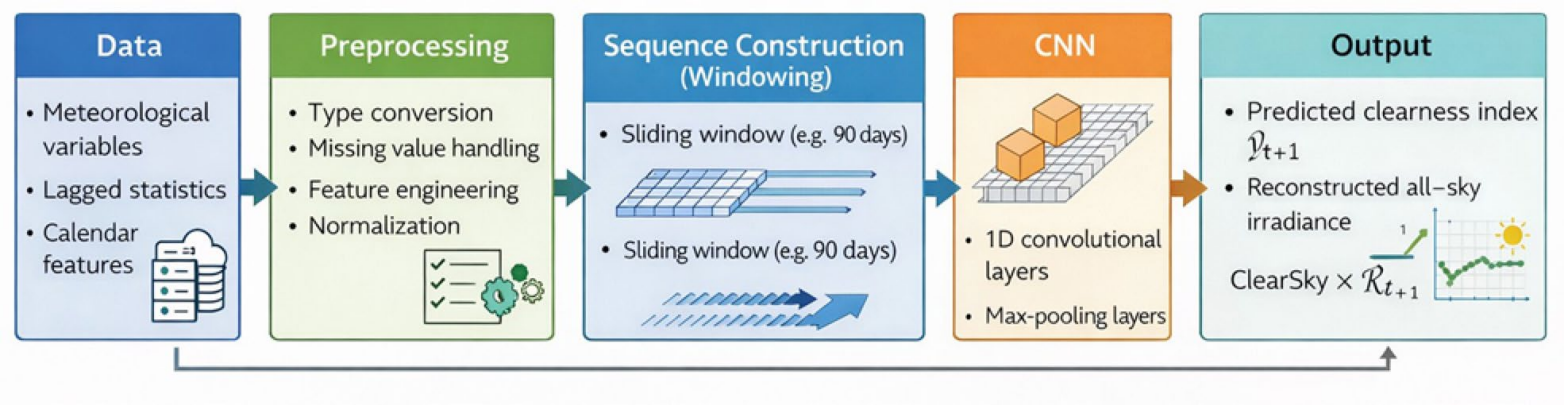
Proposed hybrid CNN–LSTM/CNN–BiLSTM workflow for solar clearness index fore- casting.

Figure 3 illustrates the complete workflow adopted in this study. The pipeline begins with data acquisition from NASA POWER, followed by cleaning (handling missing/sentinel values) and feature engineering (calendar features, lag features, and rolling statistics). Next, the multivariate time-series is normalized and transformed into supervised samples using sliding-window sequence construction. The CNN block then extracts compact local representations from the input window, after which the recurrent module (LSTM or BiLSTM) learns temporal dependencies and produces the one-step-ahead prediction. Finally, the predicted clearness index is optionally combined with clear-sky irradiance to reconstruct all-sky irradiance for practical evaluation. Presenting the work- flow in this structured form clarifies the role of each processing stage and supports reproducibility, which is strongly recommended in recent solar forecasting benchmarks^3,8,21^.

### Hybrid model design

Two hybrid architectures are evaluated under the same experimental protocol: CNN–LSTM and CNN–BiLSTM. Both models share the same convolutional front-end that transforms the raw multivariate window into a feature-rich representation, followed by a recurrent back-end that models temporal dynamics. The only difference is the recurrent unit: CNN–LSTM uses a unidirectional LSTM, whereas CNN–BiLSTM uses BiLSTM. Hybrid CNN–RNN designs are widely reported as effective for solar/PV forecasting because convolution can capture localized patterns and feature interactions while recurrence learns sequential dependence and memory effects^3,20,22,24^. In practice, such hybrids often show improved robustness under varying weather regimes compared with single-branch models^8,21^.

### CNN block for local pattern extraction

The convolutional stage applies one-dimensional convolutions along the temporal axis to learn short-range patterns, abrupt changes, and cross-variable interactions among climatic predictors. Convolution layers are well suited for multivariate time-series because their shared weights enable efficient detection of repeated local motifs, and pooling operations can reduce sensitivity to noise^20–22^. In the proposed framework, each convolution stage is followed by a nonlinear activa- tion (ReLU) and a downsampling operation (max-pooling) to compress the temporal representation.

while retaining salient responses. Dropout is used as a regularization mechanism to reduce over- fitting, which is particularly important when training deep models on limited site-specific data^3,17^. The CNN output remains sequential (i.e., a compressed time-series representation) and is forwarded to the recurrent block.

### Recurrent block for temporal modeling

The recurrent stage learns temporal dependencies using gated memory units that can capture persistence, seasonal trends, and delayed effects across the input window. Specifically:

- **CNN–LSTM**: models forward-time dynamics within the look-back sequence, which is con- sistent with realistic forecasting where future observations are unavailable at inference time^50,51^.
- **CNN–BiLSTM**: processes the same input window in both forward and backward direc- tions to enrich contextual encoding and potentially strengthen representation under complex meteorological transitions^19,23,52^.

**Fig. 4:**
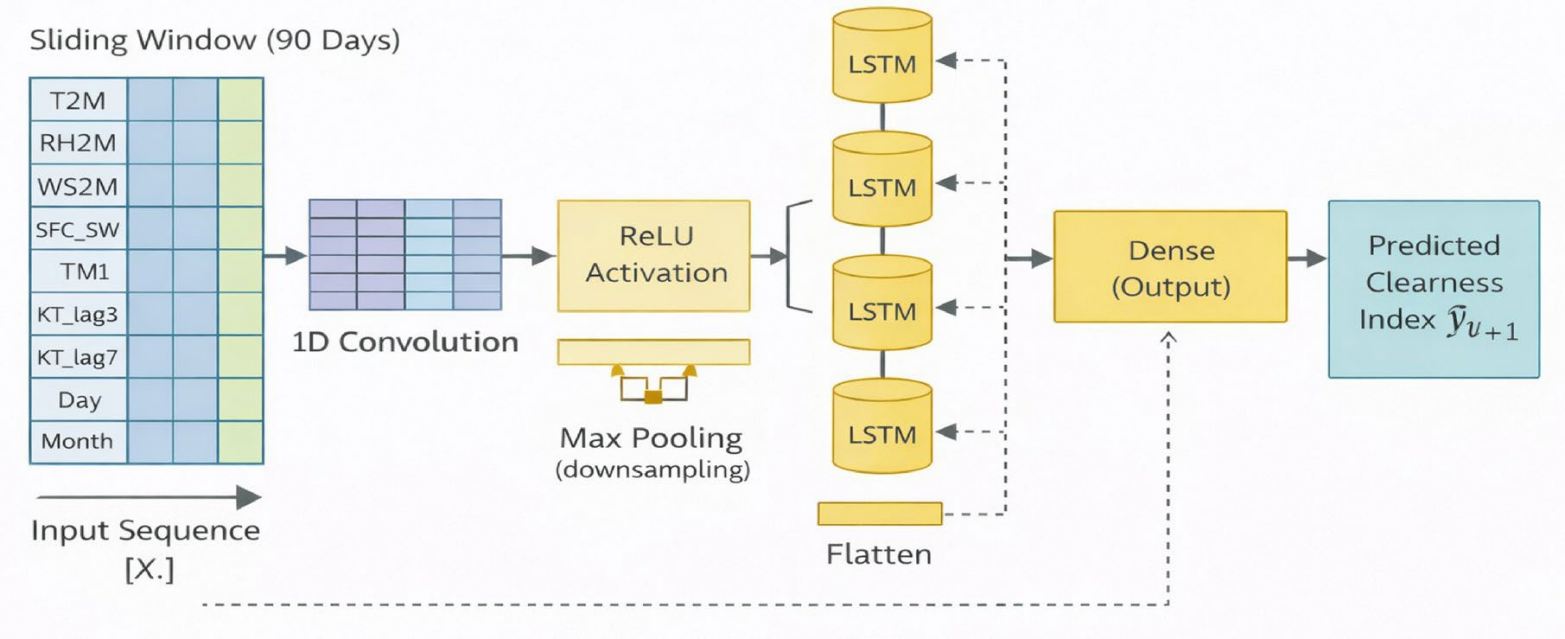
Architecture of the proposed CNN–LSTM model for one-step-ahead clearness index forecasting.

**Fig. 5:**
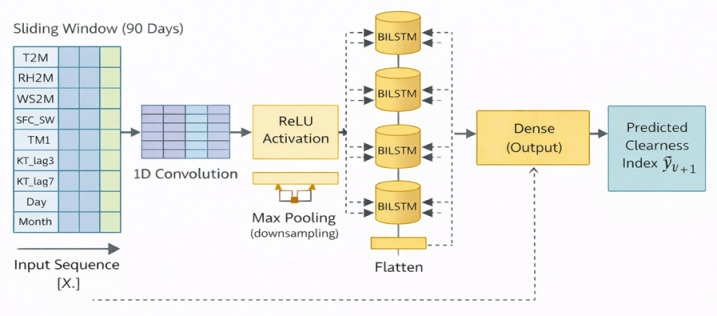
Architecture of the proposed CNN–BiLSTM model for one-step-ahead clearness index forecasting.

As shown in Fig. 4, the CNN–LSTM design feeds the CNN-extracted sequence representation into an LSTM layer that propagates information only in the forward temporal direction. This structure is appropriate for forecasting because it mirrors real deployment, where only past observations are available when predicting the next time step. In contrast, Fig. 5 illustrates that the CNN–BiLSTM variant replaces the unidirectional LSTM with a bidirectional layer, which encodes the input window using two passes (forward and backward) before combining the representations. While this can enhance feature extraction within the window, its advantage in strict forward-looking forecasting is not guaranteed and may depend on feature quality, window length, and regime variability^23,24^.

Finally, in both architectures, a Dense output layer maps the learned temporal representation to a single continuous estimate$\hat{y}_{{t + 1}}$.

### Loss function and optimization

Model learning is performed by minimizing the mean squared error (MSE) between predictions and ground truth:

$$L = \frac{1}{N}\sum_{i=1}^{N}(y_i-\hat{y}_i)^2$$

where *N* is the number of training samples. The Adam optimizer is used due to its adaptive learning-rate updates and stable convergence characteristics in deep regression settings, which is commonly reported in solar and PV forecasting studies^17,24,51^.

### Training configuration

To ensure fairness, both CNN–LSTM and CNN–BiLSTM are trained with identical hyperparame- ters and training protocol:

- **Batch size**: 32.
- **Maximum epochs**: 50.
- **Validation split**: 0.1 from the training set.
- **Reproducibility**: fixed random seeds for NumPy and TensorFlow.

Using the same data split, normalization approach, and training schedule reduces confounding factors so that performance differences primarily reflect the model architecture rather than experi- mental variation^3,43^.

### Optimal hyperparameter settings

The best hyper parameters for training the tested deep learning model. The models summarised in Table 1. These values contain the length of the sequence, The optimization settings, convolutional configuration, recurrent units, regularization Parameters and callback settings during model development. Hyperparameters were chosen with a literature-guided and validation-aware approach, instead of a held-out test set. The 90-day look-back window was selected to maintain the meteorological and seasonal context, while the filter sizes and number of recurrent-units, dropout rate, and learning rate in the CNN and the callback patience were set to values consistent with the typical CNN–RNN solar forecasts listed in the literature^8,21,22^. The early stopping and learning-rate reduction were monitored on the validation split to prevent overfitting and information leakage when using test-set.

**Table 1:** Hyperparameter settings used for training the evaluated models.

Hyperparameter	Value
Look-back window	90
Batch size	32
Maximum epochs	50
Validation split	0.1
Optimizer	Adam
Loss function	Mean Squared Error (MSE)
CNN filters	64, 32
Kernel size	3
Pooling type	MaxPooling1D
Pooling size	2
Recurrent units	50
Dropout rate	0.2
Learning rate	0.001
EarlyStopping patience	10
ReduceLROnPlateau patience	5
ReduceLROnPlateau factor	0.5
Activation function	ReLU
Output layer	Dense(1)

### Learning-rate scheduling and early stopping

Two callback-based controls are employed to improve training stability and generalization:

- **ReduceLROnPlateau**: decreases the learning rate when validation loss saturates, enabling finer updates near local minima and improving convergence behavior^17,22^.
- **EarlyStopping**: terminates training if validation loss does not improve for a defined patience period and restores the best weights observed during training, which helps prevent overfitting^3,51^.

Such training strategies are commonly used in deep learning-based solar forecasting to reduce unnecessary training epochs and to obtain models that generalize better to unseen test periods^16,17^.

### Evaluation metrics

Mean Absolute Error (MAE), Root Mean Squared Error (RMSE), coefficient of determination (R2), Explained Variance Score (EVS), Willmott index (WI), Absolute Percentage Bias (APB) and Skill Score (SCORE) were used to evaluate model performance on a hold-out chronological test set (a persistence reference was provided when available). The average absolute error (MAE), the root mean square error (RMSE), explained variability (R2, EVS), agreement (WI), systematic bias (APB), and model error compared to a reference baseline (Skill Score) are evaluated. In addition to the traditional measures of forecasting error, these complements offer a more comprehensive assessment of forecasting performance^1,20,35–41,43,50^. The other assessment indicators are:

$$WI = 1 - \frac{{\mathop \sum \nolimits_{{i = 1}}^{n} (P_{i} - O_{i} )^{2} }}{{\mathop \sum \nolimits_{{i = 1}}^{n} \left( {{\mid }P_{i} - \mathop O\limits^{`} {\mid } + {\mid }O_{i} - \mathop O\limits^{`} {\mid }} \right)^{2} }}$$

$$APB = {\mid }\frac{{100 \times \mathop \sum \nolimits_{{i = 1}}^{n} (P_{i} - O_{i} )}}{{\mathop \sum \nolimits_{{i = 1}}^{n} O_{i} }}$$

$$EVS = 1 - \frac{{Var\left( {O - P} \right)}}{{Var\left( O \right)}}$$

$$Skill{\mathrm{~}}Score = 1 - \frac{{Error_{{model}} }}{{Error_{{reference}} }}$$

where Oi represents the observed value, Pi represents the predicted value, Ō denotes the mean of observed values, n is the number of test samples, Errormodel is the error of the proposed model, and Errorreference is the error of the baseline/reference model.

These measures strengthen the evaluation by capturing agreement, bias, variance explanation, and relative improvement in addition to MAE, RMSE, and R2. Because Skill Score depends on the reference baseline, the values are interpreted relative to the persistence configuration used in the present experiment.

## Results and discussion

 This section reports the quantitative performance of the proposed hybrid deep learning models, CNN–LSTM and CNN–BiLSTM, for one-step-ahead clearness index forecasting (ALLSKY KT). In addition to the direct ALLSKY KT prediction task, the estimated clearness index is combined with the clear-sky irradiance (CLRSKY SFC SW DWN) to reconstruct all-sky surface shortwave radiation (approx. ALLSKY SFC SW DWN). Such hybrid CNN–RNN pipelines and clear-sky-guided modeling strategies are widely used in solar forecasting to improve robustness under nonlinear and weather- driven variability^1,8,21,23^.

### Clearness index forecasting performance

The results of the extended test-set for both architectures are summarized in Table 2. CNN–LSTM achieved MAE = 0.088002, RMSE = 0.109994, R2 = 0.310014, EVS = 0.315439, WI = 0.631666, and APB = 1.893775%. CNN–BiLSTM achieved MAE = 0.101534, RMSE = 0.122399, R2 = 0.145617, EVS = 0.199761, WI = 0.526122, and APB = 5.983023%. The results indicate that CNN–LSTM outperforms CNN–BiLSTM in terms of error, explained-variance, agreement, and bias measures with the given experimental protocol. For direct ALLSKY KT forecast, however, both models have negative Skill Scores meaning that the persistence reference was hard to beat. This does not negate the comparison of CNN–LSTM and CNN–BiLSTM, but it does indicate that the clearness-index results are only good in the current dataset and should not be taken as a superior performance to good baseline forecasting methods^3,23^.

**Table 2:** Extended test-set metrics for ALLSKY KT prediction.

Metric	CNN–LSTM	CNN–BiLSTM
MAE	0.088002	0.101534
RMSE	0.109994	0.122399
R2	0.310014	0.145617
EVS	0.315439	0.199761
WI	0.631666	0.526122
APB (%)	1.893775	5.983023
Skill Score (RMSE)	-0.180479	-0.313107
Skill Score (MAE)	-0.306422	-0.506467

**Fig. 6:**
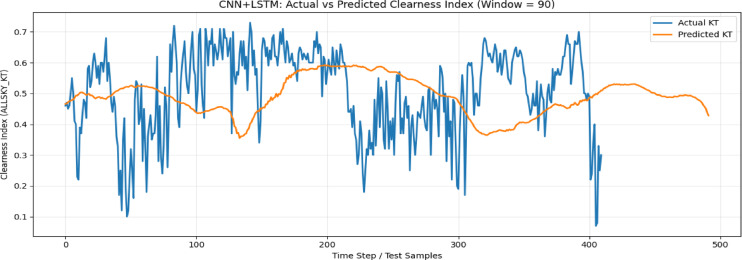
CNN–LSTM: actual vs. predicted clearness index (ALLSKY KT) on the test set.

**Fig. 7:**
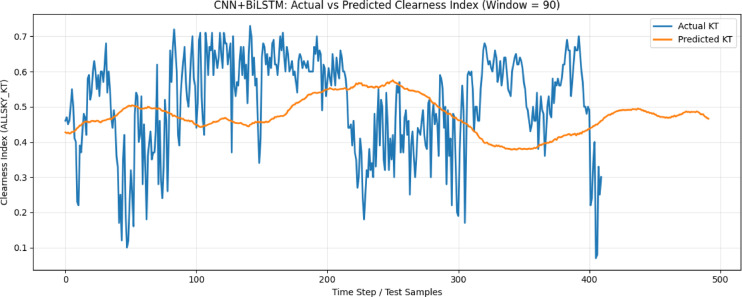
CNN–BiLSTM: actual vs. predicted clearness index (ALLSKY KT) on the test set.

Figures 6 and 7 visualize the actual and predicted ALLSKY KT series on the test interval. Both models follow the broad seasonal movement of the clearness index, but deviations are visible during sharp transitions. The moderate R2 and EVS values confirm that the models explain only part of the variance in atmospheric transparency, which is expected because sudden cloud-driven and local micro-climatic changes are difficult to infer from daily aggregated predictors alone^1,16^. Similar behavior has been reported in multivariate DL forecasting studies where performance depends strongly on site characteristics and feature availability^8,21^.

### Percentage improvement in Error

Taking CNN–BiLSTM as the comparison model and CNN–LSTM as the selected model, Table 3 reports the relative improvement obtained by CNN–LSTM. Lower values indicate better performance for MAE, RMSE, and APB, whereas higher values indicate better performance for R2, EVS, and WI.

**Table 3:** Relative improvement of CNN–LSTM over CNN–BiLSTM for direct ALLSKY KT forecasting.

Metric	CNN–BiLSTM	CNN–LSTM	Relative outcome for CNN–LSTM
MAE	0.101534	0.088002	13.33% lower error
RMSE	0.122399	0.109994	10.13% lower error
R2	0.145617	0.310014	112.90% higher explained variance
EVS	0.199761	0.315439	57.91% higher explained variance
WI	0.526122	0.631666	20.06% higher agreement
APB (%)	5.983023	1.893775	68.35% lower bias

Table 3 shows that CNN–LSTM produced lower MAE and RMSE than CNN–BiLSTM, corresponding to relative error reductions of 13.33% and 10.13%, respectively. CNN–LSTM also achieved higher R2, EVS, and WI, while reducing APB by 68.35%. These findings indicate that the unidirectional CNN–LSTM model provides a more favorable direct clearness-index forecasting result than CNN–BiLSTM in this experiment.

### Computational time and model efficiency

In addition to predictive accuracy, computational efficiency was evaluated to assess the practical feasibility of the considered models for deployment-oriented solar-radiation forecasting. Table 4 reports the trainable parameters, total training time, and average inference time per sample for all evaluated architectures. These results complement the performance metrics by highlighting the trade-off between model complexity, runtime cost, and deployment suitability.

**Table 4:** Computational complexity and runtime comparison of the evaluated models.

	Model	Trainable parameters	Training time (s)	Inference time (ms/sample)
0	CNN–LSTM	27,021	33.358	0.5152
1	CNN–BiLSTM	44,871	13.756	0.6926

Table 4 shows that CNN–LSTM provides an effective balance between model complexity and runtime efficiency. In comparison, CNN–BiLSTM is more computationally expensive because of its larger parameter count and higher inference time. These findings suggest that CNN–LSTM is the more practical model for deployment-oriented solar-radiation forecasting, as it offers slightly better predictive performance together with lower computational cost.

### Feature importance and input relevance analysis

Feature relevance was examined using the correlation heatmap, engineered temporal variables, and domain interpretation of meteorological drivers. Radiation-related inputs such as CLRSKY SFC SW DWN provide the deterministic clear-sky envelope, while lagged KT variables and rolling statistics represent recent atmospheric transparency and local variability. Temperature, humidity, precipitation, pressure, and wind speed contribute complementary information associated with cloud formation, atmospheric attenuation, and weather transitions^1,4,16,17^.

This analysis indicates that the model benefits from both physical radiation information and short-term temporal descriptors. Nevertheless, because CNN–LSTM and CNN–BiLSTM are nonlinear sequence models, correlation-based analysis should be interpreted as input relevance rather than definitive causal feature importance. Future work should extend this analysis using permutation importance, ablation experiments, SHAP-based temporal attribution, or attention-based weighting to quantify feature contributions more rigorously.

### Training and validation curves

**Fig. 8:**
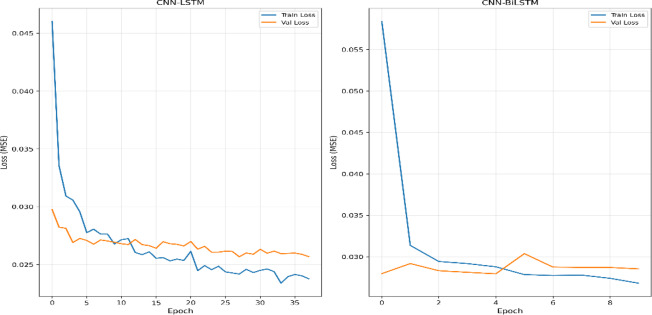
Training and validation loss curves for CNN–LSTM, CNN–BiLSTM.

Figure 8 presents the training and validation loss curves for CNN–LSTM and CNN–BiLSTM. Both models exhibit stable learning behavior during training, with training and validation losses decreasing as the epochs progress. The CNN–LSTM curve shows a smoother and more gradual decline, with only a limited gap between training and validation loss, indicating consistent convergence and good generalization. The CNN–BiLSTM model also converges appropriately, although it reaches its optimal point in fewer epochs due to early stopping once validation improvement becomes marginal. Overall, the closeness of the training and validation curves in both cases suggests that the adopted regularization strategy, dropout, and callback settings were effective in reducing overfitting and supporting reliable model learning.

### Reconstructed solar irradiance results

To assess practical irradiance prediction, all-sky solar irradiance was reconstructed by combining the predicted clearness index with clear-sky irradiance:

$$Solar(t) = ClearSky(t) \times \widehat{{KT}}(t),$$

where CLRSKY SFC SW DWN provides the clear-sky component and *KT* (*t*) is the predicted clearness index. The reconstructed irradiance results show strong agreement with observed values, achieving

**Fig. 9:**
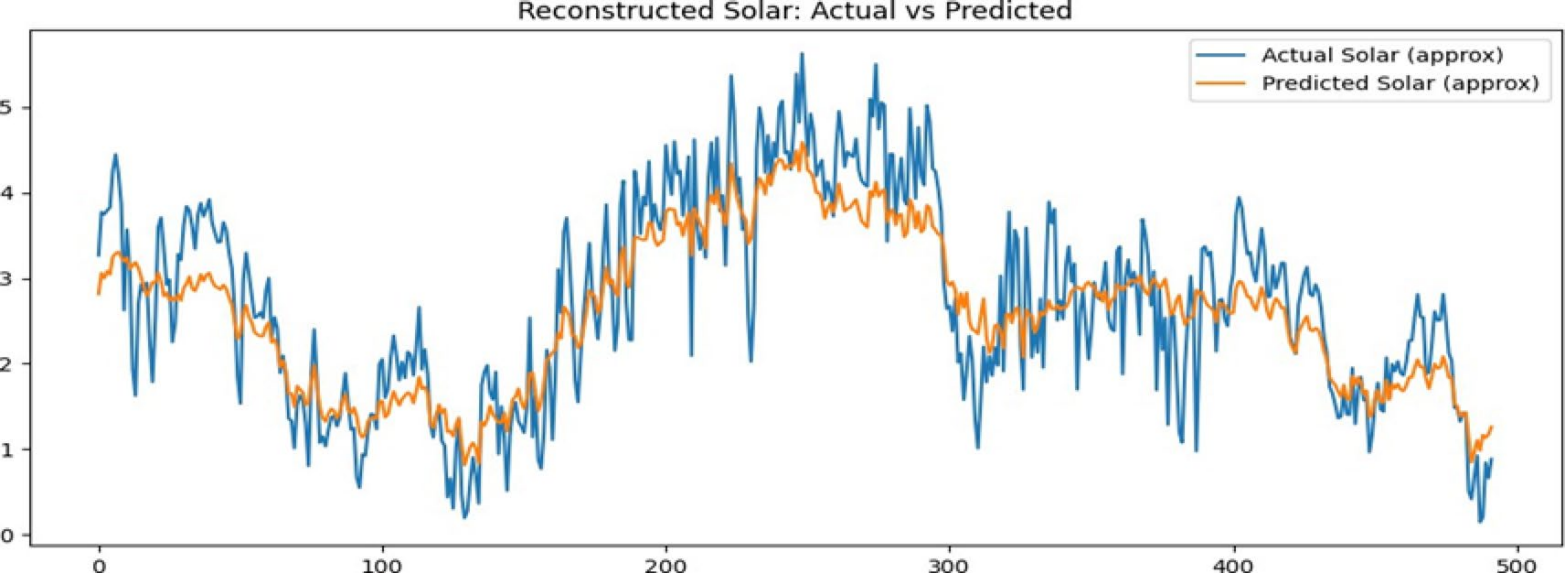
Reconstructed solar irradiance: actual vs. predicted (approx. ALLSKY SFC SW DWN).

MAE = 0.435277, RMSE = 0.541692, R2 = 0.788374, EVS = 0.796465, WI = 0.929889, APB = 3.895085%, Skill Score (RMSE) = 0.045503, and Skill Score (MAE) = -0.027347 on the aligned test period (Table 5). The higher R2, EVS, and WI values suggest that clear-sky-guided reconstruction provides stronger practical irradiance agreement than direct clearness-index forecasting by combining learned atmospheric attenuation with deterministic clear-sky radiation^1,23,42^. The positive RMSE-based Skill Score indicates a modest improvement over the selected reference baseline for squared-error behavior, whereas the slightly negative MAE-based Skill Score shows that the baseline remains competitive for average absolute error. Figure 9 confirms that reconstructed predictions follow the dominant irradiance dynamics closely, with most mismatches occurring near abrupt peaks and dips where rapid cloud movement and transient events dominate^16^.

**Table 5:** Extended reconstructed all-sky solar irradiance (approx. ALLSKY SFC SW DWN) performance.

Metric	Reconstructed Solar Irradiance
MAE	0.435277
RMSE	0.541692
R2	0.788374
EVS	0.796465
WI	0.929889
APB (%)	3.895085
Skill Score (RMSE)	0.045503
Skill Score (MAE)	-0.027347

### Uncertainty analysis

To address uncertainty in the deterministic forecasts, an approximate error-band analysis was derived from test-set RMSE values. Assuming residuals are centered around zero, a simple 95% error range can be approximated as ± 1.96 × RMSE. Under this assumption, the CNN–LSTM clearness-index forecast corresponds to an approximate error band of ± 0.216, while CNN–BiLSTM corresponds to ± 0.240. For reconstructed all-sky irradiance, the corresponding approximate band is ± 1.062 in the reported irradiance scale.

These intervals should be interpreted only as descriptive uncertainty indicators rather than formal probabilistic prediction intervals. More rigorous uncertainty quantification should be performed in future work using quantile regression, Bayesian neural networks, Monte Carlo dropout, ensemble forecasting, or kernel-density-based prediction intervals, as recommended in recent energy-demand forecasting studies^41^.

### Statistical validation of results

To further examine the consistency of the comparative results, a Wilcoxon signed-rank test was performed on fold-wise RMSE values obtained from identical time-series cross-validation splits for CNN–LSTM and CNN–BiLSTM. Table 6 presents the RMSE values across the five folds. As observed, CNN–LSTM achieved lower RMSE in four out of five folds, indicating a slight empirical advantage over CNN–BiLSTM. However, the Wilcoxon signed-rank test yielded a statistic of 2.0 with a p-value of 0.1875, which is greater than the 0.05 significance level. This indicates that the observed performance difference between the two models was not statistically significant under the present experimental setting. Therefore, CNN–LSTM may be regarded as a practically favorable model due to its slightly better predictive performance and computational efficiency, although statistical superiority over CNN–BiLSTM could not be established.

**Table 6:** Fold-wise RMSE comparison between CNN–LSTM and CNN–BiLSTM.

Fold		CNN–LSTM RMSE	CNN–BiLSTM RMSE
1		0.115637	0.152894
2		0.126190	0.137429
3		0.151289	0.147839
4		0.127671	0.141089
5		0.126508	0.128942

### Discussion and implications

Overall, CNN–LSTM outperformed CNN–BiLSTM for direct clearness-index prediction under identical training conditions, with lower MAE/RMSE, higher R2, higher EVS, higher WI, lower APB, lower parameter count, and lower inference time. However, the negative Skill Scores for both direct ALLSKY KT models indicate that the persistence reference remained a strong baseline and was not outperformed in this configuration. Therefore, the findings support CNN–LSTM as the more practical model among the two tested deep hybrids, but they do not establish state-of-the-art forecasting superiority. Prediction quality appears to depend strongly on feature informativeness, abrupt weather-driven regime shifts, and the stability of preprocessing and windowing choices^1,3^. Future benchmarking should include persistence, seasonal persistence, gradient-boosted trees, CNN–GRU, attention-based models, and transformer architectures, together with uncertainty-aware and feature-attribution methods^8,21,42–55^.

## Conclusion and future work

### Conclusion

The present work provided a controlled and reproducible comparative analysis of CNN–LSTM and CNN–BiLSTM models for solar radiation forecasting for one-step ahead prediction from multi-variate meteorological inputs. Under the same experimental protocol, CNN–LSTM achieved better direct clearness-index prediction (MAE = 0.088002, RMSE = 0.109994, R2 = 0.310014, EVS = 0.315439, WI = 0.631666, APB = 1.893775%) than CNN–BiLSTM (MAE = 0.101534, RMSE = 0.122399, R2 = 0.145617, EVS = 0.199761, WI = 0.526122, APB = 5.983023%). But the persistence baseline was still hard to beat, as seen in the negative Skill Scores for the direct clearness-index predictions. Reconstructing all-sky irradiance from predicted clearness index and clear-sky irradiance improved agreement with observations (MAE = 0.435277, RMSE = 0.541692, R2 = 0.788374, EVS = 0.796465, WI = 0.929889, APB = 3.895085%). The results indicate that CNN–LSTM is more feasible for achieving a compromise between accuracy, interpretability, and computational efficiency among the two architectures considered here, and a more comprehensive evaluation with stronger baselines, more sites, and formal probabilistic forecasting will be needed to make general statements with respect to different sites and forecast horizons.

### Limitations

There are a number of caveats to be noted. Only a single geographic location, a fixed historical time period and daily-resolution data have been analyzed, and performance may vary on the finer timescales, geographic locations and climates. Cloud-cover, aerosol, satellite and sky-image descriptors are not included in the current feature set and may be significant during sudden changes in the atmosphere. The uncertainty analysis is also approximate, and should be expanded with formal probabilistic forecasting procedures.

### Future work

Future research should investigate more baseline models such as persistence and seasonal-persistence baselines, gradient-boosted trees, CNN–GRU, attention-based recurrent networks, and transformer models under the same pipeline for benchmarking. Future work should also explore alternative weather/cloud characteristics, longer forecast horizons, and larger selection of baseline clearness-index Skill Scores, given that the directly-calculated Skill Scores were negative with respect to the chosen persistence baseline. Other validation steps such as using ensemble prediction, quantile loss-based formal prediction intervals, Bayesian inference, MC dropout, or ablation and permutation importance of features should also be implemented.

## Data Availability

The datasets used and/or analysed during the current study available from the corresponding author on reasonable request.

## References

[1] Assaf, A. M. et al. A review on neural network based models for short term solar irradiance forecasting. *Appl. Sci.* **13** (14), 8332 (2023).

[2] Ajith, M. et al. Deep learning algorithms for very short term solar irradiance forecasting: A comparative study. *Renew. Sustain. Energy Rev.* **173**, 113078 (2023).

[3] Rajagukguk, R. A., Ramadhan, R. A. & Lee, H. J. A review on deep learning models for forecasting time series data of solar irradiance and photovoltaic power. *Energies* **13** (24), 6623 (2020).

[4] Limouni, T., Yaagoubi, R., Bouziane, K., Guissi, K. & Baali, E. H. Accurate one step and multistep forecasting of very short-term pv power using lstm-tcn model. *Renew. Energy*. **205**, 1010–1024 (2023).

[5] Elizabeth Michael, N., Mishra, M., Hasan, S. & Al-Durra, A. Short-term solar power predicting model based on multi-step cnn stacked lstm technique. *Energies* **15** (6), 2150 (2022).

[6] Khan, H. A. et al. Solar irradiance forecasting using deep learning techniques, in *Proceed- ings (MDPI)*, vol. 46, no. 1, p. 15. (2023).

[7] Agga, A., Abbou, A., Labbadi, M., Houm, Y. E. & Ali, I. H. O. CNN-LSTM: An efficient hybrid deep learning architecture for predicting short-term photovoltaic power production. *Electr. Power Syst. Res.* **208**, 107908 (2022).

[8] Lim, S. C. et al. Solar power forecasting using cnn-lstm hybrid model. *Energies* **15** (21), 8233 (2022).

[9] Babalhavaeji, A. et al. Photovoltaic generation forecasting using convolutional and recurrent neural networks. *MethodsX* **10**, 102205 (2023).37206646

[10] Bou-Rabee, M. A. et al. Bilstm network-based approach for solar irradiance forecasting in continental climate zones. *Energies* **15**, 2150 (2022).

[11] Posp´ıchal, J. et al. Solar irradiance forecasting with transformer model. *Appl. Sci.* **12**, 8852 (2022).

[12] Kang, T. et al. Solar irradiance prediction based on self-attention recursive model network. *Front. Energy Res.* **10**, 977979 (2022).

[13] Al-Ali, E. M. et al. Solar energy production forecasting based on a hybrid cnn-lstm- transformer model. *Mathematics* **11** (3), 676 (2023).

[14] Liu, J. et al. A transformer-based multimodal- learning framework using sky images for ultra-short-term solar irradiance forecasting. *Ap- plied Energy*. **342**, 121160 (2023).

[15] Xie, Q. et al. An improved ssa-bilstm-based short-term irradiance prediction model via sky images feature extraction. *Renew. Energy*. **210**, 1185–1202 (2023).

[16] Sehrawat, N., Vashisht, S. & Singh, A. Solar irradiance forecasting models using machine learning techniques and digital twin: A case study with comparison. *Int. J. Intell. Networks*. **4**, 90–102 (2023).

[17] Neshat, M. et al. Short-term solar radiation forecasting using hybrid deep residual learning and gated lstm recurrent network with differential covariance matrix adaptation evolution strategy, *Energy*, (2023).

[18] Srivastava, S. & Lessmann, S. A comparative study of LSTM neural networks in forecasting day-ahead global horizontal irradiance with satellite data. *Sol. Energy*. **162**, 232–247 (2018).

[19] Brahma, B. & Wadhvani, R. Solar irradiance forecasting based on deep learning method- ologies and multi-site data. *Symmetry* **12** (11), 1830 (2020).

[20] Kumari, P. & Toshniwal, D. Long short term memory–convolutional neural network based deep hybrid approach for solar irradiance forecasting. *Appl. Energy*. **295**, 117061 (2021).

[21] Agga, A. et al. Cnn–lstm: An efficient hybrid deep learning architecture for predicting short-term photovoltaic power production, *Energy Build.*, (2022).

[22] Qu, J., Qian, Z. & Pei, Y. Day-ahead hourly photovoltaic power forecasting using attention- based cnn-lstm neural network embedded with multiple relevant and target variables prediction pattern. *Energy* **232**, 120996 (2021).

[23] Bou-Rabee, M. A. et al. Bilstm network-based approach for solar irradiance forecasting in continental climate zones. *Energies* **15** (6), 2226 (2022).

[24] Agga, A., Abbou, A., Labbadi, M. & Houm, Y. E. Short-term self consumption pv plant power production forecasts based on hybrid cnn-lstm, convlstm models. *Renew. Energy*. **177**, 101–112 (2021).

[25] Singla, P., Duhan, M. & Saroha, S. An ensemble method to forecast 24-h ahead solar irradiance using wavelet decomposition and bilstm deep learning network. *Earth Sci. Inf.* **15** (1), 291–306 (2022). 10.1007/s12145-021-00723-1 PMC8596364 34804244

[26] Gupta, A., Gupta, K. & Saroha, S. Short term solar irradiation forecasting using ceemdan decomposition based bilstm model optimized by genetic algorithm approach. *Int. J. Renew. Energy Dev.* **11** (3), 736–750 (2022).

[27] Gupta, P. & Singh, R. Combining a deep learning model with multivariate empirical mode decomposition for hourly global horizontal irradiance forecasting. *Renew. Energy*. **206**, 908–927 (2023).

[28] Camacho, M., Maldonado-Correa, J., Torres-Cabrera, J., Mart´ın-Mart´ınez, S. & Go´mez- La´zaro, E. Short-medium-term solar irradiance forecasting with a CEEMDAN-CNN-ATT- LSTM hybrid model using meteorological data. *Appl. Sci.* **15** (3), 1275 (2025).

[29] Kang, T. et al. Solar irradiance prediction based on self-attention recursive model network. *Front. Energy Res.*, (2022).

[30] Dong, H. et al. Multi-step solar radiation prediction using transformer. *J. Building Serv. Eng. Res. Technology*, (2024).

[31] Husein, M. et al. Towards energy efficiency: A comprehensive review of deep learning-based photovoltaic power forecasting strategies, *Heliyon*, article information available on ScienceDirect. https://www.sciencedirect.com/science/article/pii/S2405844024094507 (2024). 10.1016/j.heliyon.2024.e33419 PMC11268202 39050417

[32] Yu, J. et al. Deep learning models for pv power forecasting: Review. *Energies* **17** (16), 3973 (2024).

[33] Naveed, M. S. et al. Leveraging advanced AI algorithms with transformer-infused recurrent neural networks to optimize solar irradiance forecasting. *Fronti. Energy Res.*, (2024).

[34] Hou, X. et al. Prediction of solar irradiance using convolutional neural network and attention mechanism-based long short-term memory network with similar-day analysis, *Heliyon*, see full text for volume/pages.: https://pmc.ncbi.nlm.nih.gov/articles/PMC10660081/ (2023). 10.1016/j.heliyon.2023.e21484 PMC10660081 38027694

[35] AL-Musaylh, M. S. et al. Predicting near-real-time total water level with an artificial intelligence model based on Australia’s tidal wave energy belt dataset. *J. Ocean. Eng. Mar. Energy*. 10.1007/s40722-025-00394-w (2025).

[36] Al-Daffaiea, K., AL-Musaylh, M. S., Al-Faisal, Q. R. M. & Shallal, Q. M. Monthly exchange rate prediction based on artificial intelligence models and Iraqi Dinar against United States dollar, AIP Conference Proceedings, vol. 3232, no. 1, 030001, 10.1063/5.0236532 (2024).

[37] Zhang, Z. C., Hong, W. C. & Li, J. Electric load forecasting by hybrid self-recurrent support vector regression model with variational mode decomposition and improved cuckoo search algorithm. *IEEE Access.* **8**, 14642–14658. 10.1109/ACCESS.2020.2966712 (2020).

[38] Hong, W. C., Li, M. W., Geng, J. & Zhang, Y. Novel chaotic bat algorithm for forecasting complex motion of floating platforms. *Appl. Math. Model.* **72**, 425–443. 10.1016/j.apm.2019.03.031 (2019).

[39] Zhang, Z. C. & Hong, W. C. Electric load forecasting by complete ensemble empirical mode decomposition adaptive noise and support vector regression with quantum-based dragonfly algorithm. *Nonlinear Dyn.* **98**, 1107–1136. 10.1007/s11071-019-05252-7 (2019).

[40] Dong, Y., Zhang, Z. & Hong, W. C. A hybrid seasonal mechanism with a chaotic cuckoo search algorithm with a support vector regression model for electric load forecasting. *Energies* **11** (4), 1009. 10.3390/en11041009 (2018).

[41] Ghimire, S. et al. Integrated Multi-Head Self-Attention Transformer model for electricity demand prediction incorporating local climate variables,*Energy AI*, **14**, 100302, 10.1016/j.egyai.2023.100302. (2023).

[42] Zuo, H. M. et al. Ten-minute prediction of solar irradiance based on cloud detection and a long short-term memory (lstm) model. *Energy Rep.*, (2022).

[43] Khan, H. A. et al. Solar irradiance forecasting using deep learning techniques, in *Engineer- ing Proceedings*, mDPI Proceedings paper (Karachi case study). (2023).

[44] Xie, Q. et al. An improved ssa-bilstm-based short- term irradiance prediction model via sky images feature extraction. *Renew. Energy*. **219**, 119507 (2023).

[45] Ganvir, C., Dinesh, D., Gupta, R., Jha, S. K. & Raghuvanshi, P. K. Prediction of global horizontal irradiance based on explainable artificial intelligence, *Conf. Intell. Innov. Technol. Comput., Electr. Electron. (IITCEE)*, pp. 1–4. (2024).

[46] Gupta, R., Jha, S. K., Jha, P., Chaprana, K. & Singh, S. K. Enhancing the accuracy of global horizontal irradiance estimation model using convolutional neural network coupled with wavelet transform. *Eur Phys. J. Plus*, **139**, (2024).

[47] Alharkan, H. et al. Solar power prediction using dual stream cnn–lstm architecture. *Sensors* **23** (2), 945 (2023). 36679739 10.3390/s23020945 PMC9864442

[48] Liu, J. et al. A transformer-based multimodal-learning framework using sky images for ultra-short-term solar irradiance forecasting. *Appl. Energy*. **331**, 120437 (2023).

[49] Paletta, Q., Arbod, G. & Lasenby, J. Omnivision forecasting: Combining satellite and sky images for improved deterministic and probabilistic intra-hour solar energy predictions. *Appl. Energy*. **336**, 120818 (2023).

[50] Sharadga, H., Hajimirza, S. & Balog, R. S. Time series forecasting of solar power generation for large-scale photovoltaic plants. *Renew. Energy*. **150**, 797–807 (2020).

[51] Li, P., Zhou, K., Lu, X. & Yang, S. A hybrid deep learning model for short-term pv power forecasting. *Appl. Energy*. **259**, 114216 (2020).

[52] Dairi, A., Harrou, F., Sun, Y. & Khadraoui, S. A deep attention-driven model to forecast solar irradiance, in *IEEE International Conference on Industrial Informatics (INDIN)*, pp. 1–6. (2021).

[53] Uluocak, I. & Yavuz, H. Hybrid deep learning approaches for multi-class fault diagnosis in manufacturing systems. *Signal. Image Video Process.*10.1007/s11760-025-05036-0 (2025).

[54] Uluocak, I. A comparative evaluation of multivariate hybrid deep learning methods for airfoil noise forecasting. *Signal. Image Video Process.* **19** (art. 1340). 10.1007/s11760-025-04916-9 (2025).

[55] Uluocak, I. Hybrid deep learning models for predicting atmospheric methane concentrations: A comparative analysis through 2050. *Sci. China Earth Sci.* **68**, 2226–2236. 10.1007/s11430-024-1569-9 (2025).

